# Functional and therapeutic significance of EZH2 in urological cancers

**DOI:** 10.18632/oncotarget.16765

**Published:** 2017-03-31

**Authors:** Xiaobing Liu, Qingjian Wu, Longkun Li

**Affiliations:** ^1^ Department of Urology, Second Affiliated Hospital, Third Military Medical University, Chongqing, China

**Keywords:** EZH2, histone methyltransferase, prostate cancer, bladder cancer, kidney cancer

## Abstract

The enhancer of zeste homolog 2 (EZH2) is a core subunit of the polycomb repressor complex 2 (PRC2), which is overexpressed in numerous cancers and mutated in several others. Notably, EZH2 acts not only a critical epigenetic repressor through its role in histone methylation, it is also an activator of gene expression, acting through multiple signaling pathways in distinct cancer types. Increasing evidence suggests that EZH2 is an oncogene and is central to initiation, growth and progression of urological cancers. In this review, we highlight the critical role of EZH2 as a master regulator of tumorigenesis in the prostate, bladder and the kidney through epigenetic control of transcription as well as a modulation of various critical signaling pathways. We also discuss the promise and challenges for EZH2 inhibitors as future anticancer therapeutics, some of which are currently in clinical trials.

## INTRODUCTION

Human cancer genome sequencing has revealed that various histone modifying genes that encode chromatin regulators are frequently mutated in a wide variety of cancers [1–3]. Covalent epigenetic modifications at enhancers and promoters of genes regulate critical genomic and biological processes like gene expression and cell fate specification [3–9]. There is increasing evidence that the chromatin modifier EZH2 is associated with cancer [10–21]. EZH2 is one of the core enzymatic subunits of PRC2, a highly conserved protein complex that methylates lysine27 of histone H3 (H3K27) to promote transcriptional silencing of many genes [22–25]. EZH2 is overexpressed in many cancers. Also, many gain or loss of function EZH2 mutations have been discovered in distinct cancer types. Notably, EZH2 is not only a critical epigenetic repressor through histone methylation, but also an activator of gene expression through different pathways [26]. It is also clinically relevant in epigenetic cancer therapy and therefore many small molecule inhibitors have been developed that can specifically suppress the enzymatic activity of EZH2 [27–29]. Notably, a phase ½ clinical trial of EPZ-6438 in patients with advanced solid tumors was launched.

Urological cancers of the prostate, bladder and the kidney are among the 10 most frequent cancers in Chinese men [30]. Prostate cancer is a major health concern in the older male populations all over the world and is the sixth most common cause of cancer related deaths in the world [31]. Androgen receptor (AR) plays a critical role in the development of prostate cancer and androgen deprivation therapy (ADT) is the first line therapy for newly diagnosed prostate cancer patients [32]. Nevertheless, most patients progress to castration-resistant prostate cancer (CRPC) and even metastatic prostate cancer [33].

Bladder cancer incidence is 3 times higher among males than females [34]. Nearly 386000 newly diagnosed cases and about 150000 deaths are reported annually worldwide [31]. Also, 75% to 80% of new patients are diagnosed with superficial non-muscle invasive bladder cancer (NMIBC) [35] Renal cell carcinoma is the eighth most common cancer in the United States [36] and its incidence is steadily rising in most areas of the world [37]. Total or partial nephrectomy is the optimal primary treatment. Nevertheless, renal cell carcinoma recurs in 20-40% of patients after resection, which is associated with tumor stage and grade [38].

In this review, we highlight the transcriptional function of EZH2 in cancer and the current insights into the role of EZH2 in prostate, bladder and kidney cancer. Finally, we will review the development, translation and early clinical findings of therapeutics targeting EZH2 in cancer.

## THE FUNCTIONAL ROLE OF EZH2

**Figure 1:**
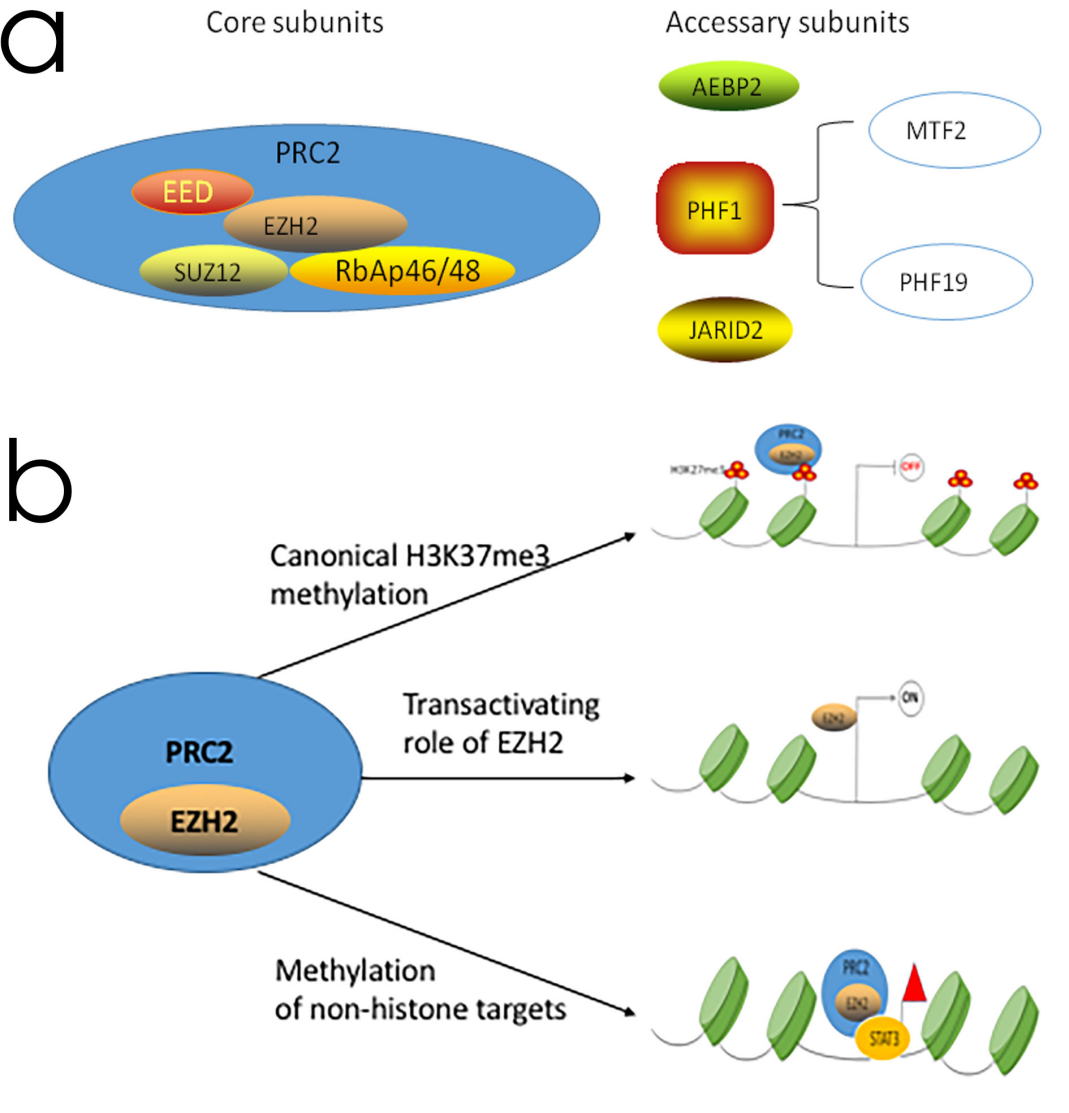
The PRC2 complex structure and the function of EZH2 in transcriptional regulation **a**. The PRC2 complex consist of four core subunits, namely, EZH2, EED, SUZ12 and RbAp46/48 and additional proteins like AEBP2, PHF1, and JARID2. **b**. The functional role of EZH2: as a subunit of PRC2, EZH2 methylates H3K27 which contributing transcriptional silencing, EZH2 also have a PRC2 independent role in transcriptional activation and can methylate a number of non-histone protein substrates. OFF and ON refer to transcriptional silencing and activation, respectively.

Human EZH2 gene is located on the long arm of chromosome 7 at 7q35 and encodes a 746 amino acid protein that is part of the PRC2 complex, which also includes SUZ12, EED, RbAp46 and RbAp48 as shown in Figure 1a [30]. PRC2 is a methyltransferase that methylates lysine 27 of histone H3 (H3K27me3) [39]. Many studies have implicated EZH2 as a key player in tumorigenesis. The role of EZH2 in cancer was first observed when it was identified as one of the top upregulated genes in aggressive prostate cancer [10]. Since then, similar findings have been reported in other human cancers including breast cancer, bladder cancer, renal cell carcinoma, etc [40–42]. In most cases, high EZH2 expression is associated with metastasis and advanced disease in each of these cancer types. Collectively, the biological function of EZH2 includes canonical H3K27 methylation, transactivation of gene expression and methylation of non-histone targets.

### Canonical H3K27 methylation

As a core subunit of PRC2, EZH2 methylates H3K27me3 that leads to transcriptional silencing. The SET domain is the catalytic subunit of EZH2 [43]. For epigenetic silencing, EZH2 complexes with EED and SUZ12, which are the two other subunits of the PRC2 complex. Given its role as a transcriptional repressor (Figure 1b), substantial efforts have been dedicated to understand the mechanism by which EZH2 drives tumor development.

#### EZH2 and cancer initiation

EZH2 is essential for self renewal in stem cells [44]. Analogous to its role in normal stem cells, EZH2 suppresses differentiation via canonical H3K27 methylation to repress lineage specifying factors [45, 46]. EZH2 is essential survival and proliferation of breast tumor initiating cells [44]. High EZH2 levels have been observed in cancer stem cell (CSC) populations isolated from primary breast cancer cells compared to normal breast cell lines [44]. Further, EZH2 activates RAF1-β-catenin signaling pathway that promotes expansion of breast tumor initiating cells. Therefore, it is hypothesized that EZH2 promotes cancer initiation by blocking differentiation [47]. However, EZH2 is also essential for differentiation programs of several distinct cancer types [48]. Therefore, the primary role of EZH2 is envisaged to include suppression of lineage specifying transcription programs in CSC and its effects on stemness and differentiation are probably secondary consequences.

#### EZH2 and tumor metastasis

EZH2 mediates silencing of FOXC1 and DNA damage repair pathways thereby driving oncogenesis [44, 49, 50]. Moreover, EZH2 promotes epithelial-mesenchymal transition (EMT) by epigenetically suppressing E-cadherin (also known as CDH1) via canonical H3K27me3 modification of its promoter [50–53], facilitated by MEK/ERK signaling [54]. Further, EZH2 interacts with HDAC1/HDAC2 and Snail to form a co-repressor complex that contributes to E-cadherin promoter repression [55]. EZH2 is also required to recruit Snail-Ring1A/B complex to the E-cadherin promoter [56]. Consequently, E-cadherin inhibition is correlated with advanced stage cancer with poor clinical outcomes [56].

#### EZH2 and tumor progression

There is increasing evidence that EZH2 promotes angiogenesis in clear renal cell carcinoma, inflammatory breast cancer, nasopharyngeal carcinoma (NPC) and glioblastomas (GBM) [13, 40, 57]. High expression levels of EZH2 and VEGF correlate with TMN stage and distant metastasis in advanced clear renal cell carcinoma [13]. In nasopharyngeal carcinoma (NPC), elevated EZH2 levels were associated with an aggressive phenotype with poor prognosis and enhanced microvessel density [18]. EZH2 also promotes angiogenesis by inhibiting miR-1/Endothelin-1, which is an autocrine regulator of endothelial cells during neovascularization. Conversely, EZH2 represses angiogenesis during hypoxia and ischemia through its hypoxia response element (HRE) [58]. In endothelial cells, hypoxia results in EZH2 overexpression that regulates two pro-angiogenic genes, eNOS and BDNF, by augmenting the abundance of H3K27me3 at their promoters. However, from a therapeutic perspective, it is not clear if the EZH2 targets are essential for all cancer types.

### Transactivating role of EZH2

Several studies have also identified a PRC2-independent role of transcriptional activation for EZH2 (Figure 1b) [59–62]. In a castration-resistant prostate cancer model, EZH2 acted as a co-activator for critical transcription factors including the androgen receptor (AR) that was independent of its transcriptional repressor function [63]. Further, EZH2 physically bridged the estrogen receptor (ER) and components of Wnt signaling to induce the gene expression in breast cancer cells [59]. EZH2 also activated NF-κB targets of NOTCH1 in breast cancer cells [61, 62].

### Methylation of non-histone targets

Another PRC2-independent role of EZH2 is methylation of non-histone targets (Figure 1b). In a castration-resistant prostate cancer model, EZH2 methylated AR and modulated AR recruitment to its target sites [63]. Further, EZH2 promoted tumorigenicity of glioblastoma stem-like cells by methylating STAT3 [64]. EZH2 also methylates non-histone substrates that are recognized by the ubiquitination machinery for degradation [65].

Therefore, the biological functions of EZH2 include epigenetic repression through histone methylation as well as transcriptional activation of genes by modulating activity of various transcription factors and other associated proteins. However, the functional significance of the non-canonical functions of EZH2 to tumorigenesis is unclear at the present.

## DYSREGULATION AND FUNCTIONAL ROLES OF EZH2 IN UROLOGY CANCER

### EZH2 and prostate cancer

**Figure 2:**
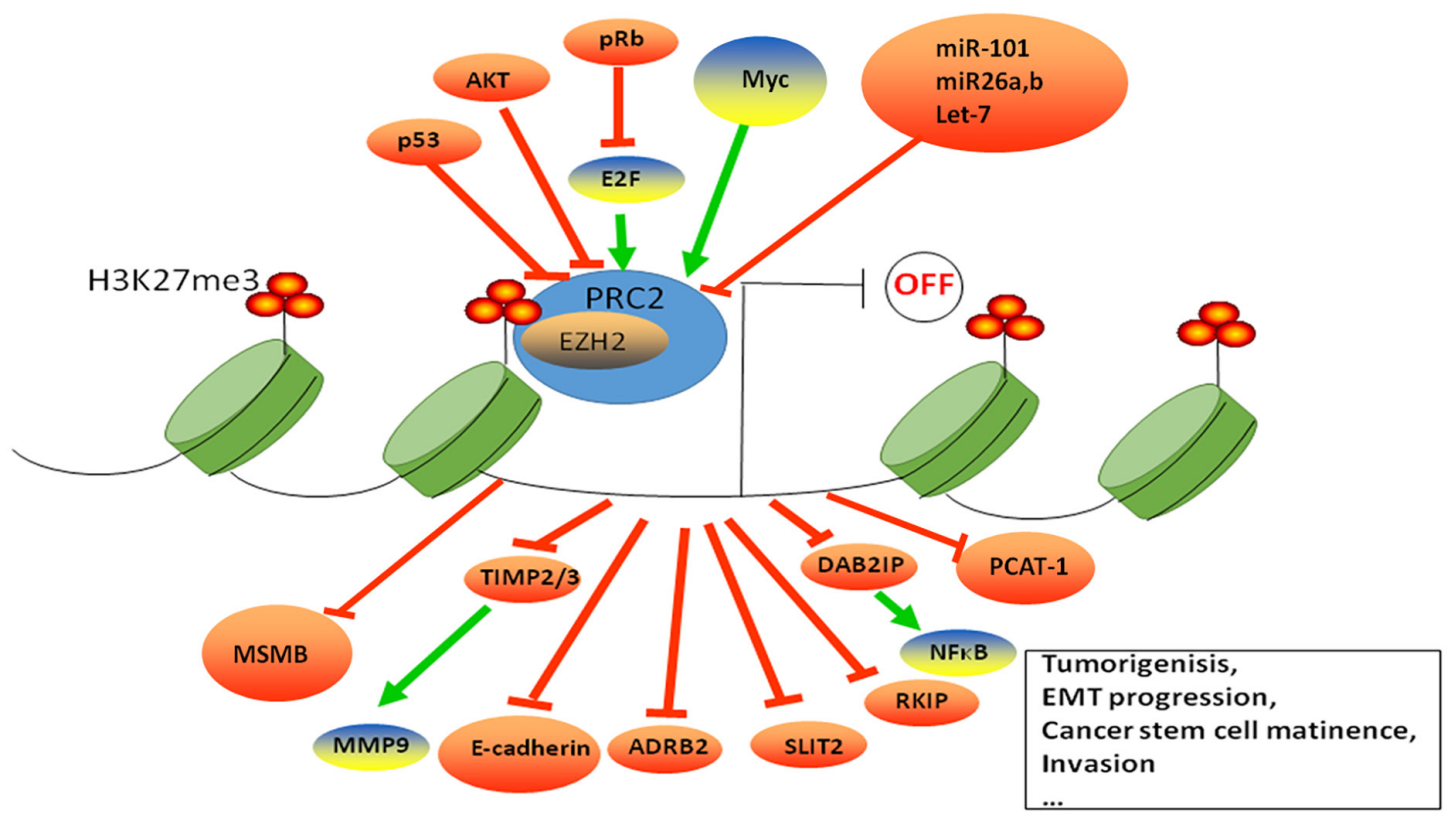
EZH2 regulation and function in prostate cancer EZH2 is the enzymatic subunit of PRC2, which catalyzes H3K23me3. EZH2 induces transcriptional silencing of tumor suppressor genes, which subsequently cause tumor initiation, growth and progression. EZH2 is regulated by E2F, p53, MYC, AKT, miR101, miR26a, miR26b and Let-7.

Varambally *et al* first demonstrated a positive association between EZH2 protein levels and prostate cancer aggressiveness [10]. Since then, many studies have highlighted the association between EZH2 expression and prostate cancer development [66–68]. Notably, EZH2 overexpression is not only associated with metastasis, but also with higher risk of recurrence after radical prostatectomy [10]. Hence, EZH2 is considered a potential diagnostic and prognostic biomarker in prostate cancer (Figure 2).

EZH2 is regulated transcriptionally, post-transcriptionally, and translationally (Figure 2). It also integrates and modulates many signaling pathways (Table 2). The E2F transcriptional factors bind to EZH2 and EED promoters and regulate their expression during E2F mediated cell proliferation via EZH2 [69]. In contrast, activated p53 suppresses EZH2 gene expression by repressing the EZH2 promoter via p21 that inactivates pRB/E2F transcriptionally [70]. Further, SKP2-TRAF6 pathway tightly regulates EZH2 expression by ubiquitination [71]. A recent study showed that a transcriptional repressor, ZFN217 interacted with EZH2 to enhance H3K23me3 levels of FPN promoter to promote prostate cancer growth [72]. The splicing factor SF3B3 stimulates inclusion of exon 14 of EZH2 that promotes proliferation [73]. The lncRNA MALAT1 interacts with the N-terminal of EZH2 to enhance migration and invasion in castration-resistant prostate cancer [74].

**Table 1:** List of downstream targets of EZH2 in urological cancer

Cancer type	Target genes	Function	Contribution to tumorigenesis	Reference
Prostate	MSMB	Inhibits MMP secretion	Proliferation and invasion	83
	DAB2IP	Inhibition of NF-kB/Ras pathway	Transformation, proliferation and invasion	75, 76
	E-cadherin	maintain epithelial cellular adhesion	invasion	92
	ADRB2	B-adrenergic signaling	Transformation and invasion	77
	SLIT2	Chemorepellent protein	Proliferation and invasion	79
	TIMP2/3	ECM degradation	Invasion	82
	PCAT-1	Transcriptional repressor lincRNA	Proliferation	84
	RKIP	Inhibition of Raf and NF-kB pathways	Invasion	85
Bladder	APAF-1	apoptosis promoting factor	Proliferation and invasion	91
	E-cadherin	maintain epithelial cellular adhesion	Invasion	92
Kidney	E-cadherin	maintain epithelial cellular adhesion	Invasion	53

**Table 2:** List of upstream targeting EZH2 in urological cancer

Cancer type	Upstream modulator	Reference
prostate	AKT, p53, E2F, Myc, miR-101,miR-26a/b, Let-7	66,77,78,86
bladder	Myc, E2F, miR-144, miR-101, miR-26a	81,82,84,87
kidney	YB-1, LncRNA MALAT1	97,98

As a histone trimethyltransferase, EZH2 represses transcription of a number of tumorigenesis and metastasis suppressor genes thereby regulating prostate cancer development. A list of direct targets of EZH2 is shown in Table 1, which indicates that EZH2 is a bonafide oncogene. Enhanced EZH2 expression suppresses DABI2P expression that is part of the Ras-NFkB signaling pathway resulting in initiation and metastasis of prostatic tumors [75, 76] . Moreover, EZH2 represses the expression of adrenergic receptor beta 2 (ADRB2), which is a critical mediator of β-adrenergic signaling that ultimately leads to cell transformation and invasion [77]. E-cadherin is another EZH2 target that mediates epithelial to mesenchymal transition [50]. Also, PSP94, SLIT2 and CDKN2A are downstream targets of EZH2 in prostate cancer that mediates tumorigenesis and metastasis. PSP94 is a suppressor of tumor growth and metastasis; SLIT2 inhibits prostate cancer cell proliferation and invasion; and CDKN2A is a critical tumor suppressor gene [78–80]. Moreover, a direct relationship between EZH2 and TIMP2/3-tissue inhibitors of metalloproteinase-results in enhanced proteolytic activity of MMP-9 in prostate cancer cells [81]. The lncRNA, DANCER represses TIMP2/3 expression by mediating the binding of EZH2 on their promoters thereby promoting prostate cancer invasiveness [82]. High expression of the EZH2 gene is also associated with low MSMB levels in metastasizing prostate cancer [83]. Meanwhile, EZH2 can repress long non-coding RNA, PCAT-1, which is a prostate specific regulator of cell proliferation [84]. In addition, Raf-1 kinase inhibitor protein (RKIP), a tumor and metastasis suppressor is repressed by EZH2. Lack of RKIP disrupts major cellular signaling pathways like Raf-1/MEK/ERK, NFkβ, and GPCR resulting in prostate cancer development and metastasis [85]. Furthermore, RNNX1, a direct target of AR is repressed by H2K27me3 and is negatively regulated by EZH2 [86]. Notably, miR-26a and miR-138a block the G1/S-phase transition in prostate cancer, independent of EZH2, via a concerted inhibition of crucial cell cycle regulators [87].

### EZH2 and bladder cancer

Many studies have indicated that there are multiple modes of regulating EZH2 that act in concert. EZH2 can be transcriptionally induced by E2F family transcription factors [69, 88]. Further, it can be regulated post-transcriptionally by the interaction with many microRNAs and long non-coding RNAs [89]. Moreover, its protein level can be modulated by ubiquitination linked degradation through PI3K-Akt phosphorylation [90]. EZH2 transcriptional activity correlates with methylation of the APAF-1 gene, which is associated with superficial transitional cell carcinoma of the bladder [91]. Further, EZH2 mediates transcriptional silencing of the tumor suppressor gene, E-cadherin [50, 92]. In addition, the pRB-E2F pathway tightly regulates EZH2 expression that promotes bladder cancer development [93]. Further, BDR4 regulates EZH2 transcription by upregulating c-Myc, thereby suggesting a novel therapeutic target in bladder cancer [94].

Several miRNAs are involved in EZH2 regulation. The microRNAs are small non-coding transcripts, 20-22 nucleotides long that participate in many fundamental biological processes including development, apoptosis, differentiation and proliferation [95]. Some like miR-101, miR-144 directly regulate EZH2 post-transcriptionally [96, 97]. In mouse fibroblasts, histone demethylase KDM2B induces expression of miR-101 that targets EZH2 [98]. A similar NDY1/KDM2B-miR101-EZH2 axis was identified in bladder cancer [99]. Meanwhile, miR144-EZH2 axis promotes bladder cell proliferation by regulating the Wnt signaling pathway [96]. Conversely, EZH2 also regulates a wide variety of miRNAs like the miR200 family and miR143 through epigenetic repression. These miRNAs regulate tumor suppressors thereby modulating tumor growth, maintain cancer stem phenotype and cancer cell invasiveness.

Several lncRNAs interact with PRC2 and facilitate access to the promoter of some target genes. LncRNAs act as scaffolds for chromatin modifying factors that alter histone markers thereby modifying gene expression [100]. The lncRNA UNMIBC physically associates with EZH2 and is associated with recurrence of primary invasive bladder cancer [101]. Further, lncRNA H19 is an enhancer that promotes bladder cancer metastasis by inhibiting E-cadherin expression through epigenetic silencing [92]. Also, the lncRNA UBC1 is physically associated with the PRC2 complex and frequently upregulated in bladder cancer [102]

### EZH2 and renal cell carcinoma

Many studies have demonstrated that EZH2 plays crucial roles in the initiation, growth and progression of renal cell carcinoma (RCC) [103–106]. Wagener *et al* suggested that EZH2 is an independent prognostic marker indicating poor cancer specific survival (CSS) in RCC [107] . Hinz *et al* demonstrated that high EZH2 levels indicated less aggressive tumor phenotypes with a favorable prognosis in RCC [12].

Many factors regulate EZH2 in regard to RCC. YB1 regulates EZH2 post-transcriptionally [108]. Long non-coding RNAs, such as HOTAIR and MALAT1, promote aggressive renal cell carcinoma by associating with EZH2 [109, 110]. MiR101 suppresses EZH2 that results in decreased renal cancer cell proliferation [111]. MiR138 induces RCC senescence by targeting EZH2 [112]. Du *et al* showed that CDH5, a chromatin remodeling factor, suppressed the expression of EZH2 [113].

Meanwhile, EZH2 enhanced proliferation and invasion of the renal cell carcinoma cell line ACHN via Wnt / β-catenin pathway [114]. Also, EZH2 positively correlated with VEGF expression [13]. Additionally, high EZH2 expression repressed E-cadherin and was associated with advanced disease state and poor survival of RCC patients [53].

## EZH2 AS A THERAPEUTIC TARGET

As discussed in previous sections, EZH2 has a critical role in tumor initiation, growth and progression in urological cancers. Further, downregulation of EZH2 demonstrates potential benefits for suppressing the urological cancers [115–120]. Therefore, there is great interest and effort to develop EZH2 specific inhibitors and multiple phase I trials are underway to analyze potential clinical benefits.

3-deazaadenosine (DZNep) has been widely used to inhibit EZH2. However, DZNep is not specific to EZH2 [27]. It depletes PRC2 proteins and inhibits histone H3K27 methylation in various cancer types [121–126]. Among the drawbacks, DZNep has a very short plasma half-life and mediates non-specific inhibition of histone methylation and is toxic in animal models [127]. Therefore, currently efforts have been directed towards developing inhibitors that are potent and specific to EZH2 to reduce toxicity and improve antitumor activities. EPZ005687 is a potent inhibitor of EZH2 that demonstrates 500 fold greater selectivity compared to other human protein methyl transferases and 50 fold more selective than EZH1 [28, 128]. GSK126 is another inhibitor with a 1000 fold more selective compared to 20 other human methyl transferases and 150 fold more selective over EZH1 [129]. And GSK343 is inhibitor with a 1000 fold over other human methyl transferases and 60 fold over EZH1 [130]. EI1 is another EZH1 inhibitor that shows >10000-fold selectivity over other methyl transferases and 90 fold more selectivity over EZH1 [131]. In all these cases, there is increased expression of PRC2 targets. Notably, many of these compounds require frequent injection. Hence, UNC1999, the first orally available inhibitor with high *in vitro* potency for wild-type and mutant EZH2 as well as EZH1 is preferred [132]. Currently, another EZH2 inhibitor, EPZ-6438 has been developed that has better pharmacokinetic properties than EPZ005687 and better oral bioavailability [133]. In June 2013, a phase 1/2 clinical trial of EPZ7438 was launched in patients with either advanced solid tumor or B cell lymphomas (NCT01897571). In addition, a biologically active biphenolic compound, honokiol was isolated from *Magnolia officinalis* that inhibited human urinary bladder cancer (UBC) cell proliferation, migration and invasion by downregulating EZH2 [117]. EZH2 can also be inhibited by disrupting PRC2 stability through the use of a peptide known as stabilized alpha-helix of EZH2(SAH-EZH2) that is derived from the domain of EZH2 that interacts with EED [134].

Meanwhile, reports of therapy resistance to EZH2 inhibitors have also been reported. In a cell line model of acquired resistance to EZH2 inhibitor EPZ-6438, two novel secondary mutations of EZH2 (Y111L and Y661D) were identified following prolonged exposure to EZH2 inhibitors that were associated with therapy resistance [135]. A combination of GSK126 and DZNep significantly increased cell death *in vitro* in murine and human prostate cancer cell lines [136]. Recent data also suggests that concomitant administration of small molecule inhibitors of EZH2 significantly increases the antitumor efficacy of conventional chemo-and radiotherapies in CRPC [115].

In summary, the development of EZH2 inhibitors for cancer therapy is in early stages and there have been reports of resistance that need to be addressed. Further studies are ongoing for potential combination therapy that includes use of EZH2 inhibitors.

## CONCLUSIONS, QUESTIONS AND FUTURE DIRECTIONS

In conclusion, we have reviewed both clinical and basic studies that clearly indicate that EZH2 is an oncogene in urological cancers. Whole genome analysis has indicated that the downstream targets of EZH2 are cancer specific [137]. Since EZH2 has a dual role in epigenetic repression and signaling activation, it is interesting to investigate the consequence of gain of function EZH2 mutations towards cancer development in terms of PRC2 dependency. Compared to its epigenetic role, the signaling pathways involving EZH2 need to be further studied in detail.

EZH2 was originally discovered as a regulator of body patterning in fruit flies.[138] As of now, it is recognized as a critical driver of cancer initiation, growth and progression through transcriptional regulation of chromatin structure. Future investigations into the role of EZH2 in urological cancers would require application of advanced techniques including microarrays and RNAseq. Newer technological advances will potentially pave the way for novel EZH2 inhibitors for therapeutic use in near future.

## References

[1] Dalgliesh GL, Furge K, Greenman C, Chen L, Bignell G, Butler A, Davies H, Edkins S, Hardy C, Latimer C, Teague J, Andrews J, Barthorpe S (2010). Systematic sequencing of renal carcinoma reveals inactivation of histone modifying genes. Nature.

[2] Gui Y, Guo G, Huang Y, Hu X, Tang A, Gao S, Wu R, Chen C, Li X, Zhou L, He M, Li Z, Sun X (2011). Frequent mutations of chromatin remodeling genes in transitional cell carcinoma of the bladder. Nature genetics.

[3] Ho AS, Kannan K, Roy DM, Morris LG, Ganly I, Katabi N, Ramaswami D, Walsh LA, Eng S, Huse JT, Zhang J, Dolgalev I, Huberman K (2013). The mutational landscape of adenoid cystic carcinoma. Nature genetics.

[4] Mikkelsen TS, Ku M, Jaffe DB, Issac B, Lieberman E, Giannoukos G, Alvarez P, Brockman W, Kim TK, Koche RP, Lee W, Mendenhall E, O’Donovan A (2007). Genome-wide maps of chromatin state in pluripotent and lineage-committed cells. Nature.

[5] Orkin SH, Hochedlinger K (2011). Chromatin connections to pluripotency and cellular reprogramming. Cell.

[6] Creyghton MP, Cheng AW, Welstead GG, Kooistra T, Carey BW, Steine EJ, Hanna J, Lodato MA, Frampton GM, Sharp PA, Boyer LA, Young RA, Jaenisch R (2010). Histone H3K27ac separates active from poised enhancers and predicts developmental state. Proc Natl Acad Sci U S A.

[7] Bernstein BE, Mikkelsen TS, Xie X, Kamal M, Huebert DJ, Cuff J, Fry B, Meissner A, Wernig M, Plath K, Jaenisch R, Wagschal A, Feil R (2006). A bivalent chromatin structure marks key developmental genes in embryonic stem cells. Cell.

[8] Cui K, Zang C, Roh TY, Schones DE, Childs RW, Peng W, Zhao K (2009). Chromatin signatures in multipotent human hematopoietic stem cells indicate the fate of bivalent genes during differentiation. Cell Stem Cell.

[9] Rada-Iglesias A, Bajpai R, Swigut T, Brugmann SA, Flynn RA, Wysocka J (2011). A unique chromatin signature uncovers early developmental enhancers in humans. Nature.

[10] Varambally S, Dhanasekaran SM, Zhou M, Barrette TR, Kumar-Sinha C, Sanda MG, Ghosh D, Pienta KJ, Sewalt RG, Otte AP, Rubin MA, Chinnaiyan AM (2002). The polycomb group protein EZH2 is involved in progression of prostate cancer. Nature.

[11] Raman JD, Mongan NP, Tickoo SK, Boorjian SA, Scherr DS, Gudas LJ (2005). Increased expression of the polycomb group gene, EZH2, in transitional cell carcinoma of the bladder. Clin Cancer Res.

[12] Hinz S, Weikert S, Magheli A, Hoffmann M, Engers R, Miller K, Kempkensteffen C (2009). Expression profile of the polycomb group protein enhancer of Zeste homologue 2 and its prognostic relevance in renal cell carcinoma. J Urol.

[13] Xu ZQ, Zhang L, Gao BS, Wan YG, Zhang XH, Chen B, Wang YT, Sun N, Fu YW (2015). EZH2 promotes tumor progression by increasing VEGF expression in clear cell renal cell carcinoma. Clin Transl Oncol.

[14] Kim KH, Kim L, Choi SJ, Han JY, Kim JM, Chu YC, Kim YM, Park IS, Lim JH (2014). The clinicopathological significance of epithelial mesenchymal transition associated protein expression in head and neck squamous cell carcinoma. Korean journal of pathology.

[15] Ahani N, Shirkoohi R, Rokouei M, Alipour Eskandani M, Nikravesh A (2014). Overexpression of enhancer of zeste human homolog 2 (EZH2) gene in human cytomegalovirus positive glioblastoma multiforme tissues. Medical oncology.

[16] Chisholm KM, Wan Y, Li R, Montgomery KD, Chang HY, West RB (2012). Detection of long non-coding RNA in archival tissue: correlation with polycomb protein expression in primary and metastatic breast carcinoma. PLoS One.

[17] Reijm EA, Timmermans AM, Look MP, Meijer-van Gelder ME, Stobbe CK, van Deurzen CH, Martens JW, Sleijfer S, Foekens JA, Berns PM, Jansen MP (2014). High protein expression of EZH2 is related to unfavorable outcome to tamoxifen in metastatic breast cancer. Annals of oncology.

[18] Lu J, Zhao FP, Peng Z, Zhang MW, Lin SX, Liang BJ, Zhang B, Liu X, Wang L, Li G, Tian WD, Peng Y, He ML (2014). EZH2 promotes angiogenesis through inhibition of miR-1/Endothelin-1 axis in nasopharyngeal carcinoma. Oncotarget.

[19] Deb G, Thakur VS, Gupta S (2013). Multifaceted role of EZH2 in breast and prostate tumorigenesis: epigenetics and beyond. Epigenetics.

[20] Herviou L, Cavalli G, Cartron G, Klein B, Moreaux J (2016). EZH2 in normal hematopoiesis and hematological malignancies. Oncotarget.

[21] Warrick JI, Raman JD, Kaag M, Bruggeman T, Cates J, Clark P, DeGraff DJ (2016). Enhancer of zeste homolog 2 (EZH2) expression in bladder cancer. Urologic oncology.

[22] Margueron R, Reinberg D (2011). The Polycomb complex PRC2 and its mark in life. Nature.

[23] Di Croce L, Helin K (2013). Transcriptional regulation by Polycomb group proteins. Nature structural & molecular biology.

[24] Zhang J, Bardot E, Ezhkova E (2012). Epigenetic regulation of skin: focus on the Polycomb complex. Cellular and molecular life sciences.

[25] Muller J, Hart CM, Francis NJ, Vargas ML, Sengupta A, Wild B, Miller EL, O’Connor MB, Kingston RE, Simon JA (2002). Histone methyltransferase activity of a Drosophila Polycomb group repressor complex. Cell.

[26] Deb G, Singh AK, Gupta S (2014). EZH2: not EZHY (easy) to deal. Molecular cancer research.

[27] Glazer RI, Hartman KD, Knode MC, Richard MM, Chiang PK, Tseng CK, Marquez VE (1986). 3-Deazaneplanocin: a new and potent inhibitor of S-adenosylhomocysteine hydrolase and its effects on human promyelocytic leukemia cell line HL-60. Biochemical and biophysical research communications.

[28] Knutson SK, Wigle TJ, Warholic NM, Sneeringer CJ, Allain CJ, Klaus CR, Sacks JD, Raimondi A, Majer CR, Song J, Scott MP, Jin L, Smith JJ (2012). A selective inhibitor of EZH2 blocks H3K27 methylation and kills mutant lymphoma cells. Nature chemical biology.

[29] Knutson SK, Kawano S, Minoshima Y, Warholic NM, Huang KC, Xiao Y, Kadowaki T, Uesugi M, Kuznetsov G, Kumar N, Wigle TJ, Klaus CR, Allain CJ (2014). Selective inhibition of EZH2 by EPZ-6438 leads to potent antitumor activity in EZH2-mutant non-Hodgkin lymphoma. Molecular cancer therapeutics.

[30] Chen W, Zheng R, Baade PD, Zhang S, Zeng H, Bray F, Jemal A, Yu XQ, He J (2016). Cancer statistics in China, 2015. CA Cancer J Clin.

[31] Ferlay J, Shin HR, Bray F, Forman D, Mathers C, Parkin DM (2010). Estimates of worldwide burden of cancer in 2008: GLOBOCAN 2008. Int J Cancer.

[32] Malik R, Khan AP, Asangani IA, Cieslik M, Prensner JR, Wang X, Iyer MK, Jiang X, Borkin D, Escara-Wilke J, Stender R, Wu YM, Niknafs YS (2015). Targeting the MLL complex in castration-resistant prostate cancer. Nature medicine.

[33] Kahn B, Collazo J, Kyprianou N (2014). Androgen receptor as a driver of therapeutic resistance in advanced prostate cancer. International journal of biological sciences.

[34] Ploeg M, Aben KK, Kiemeney LA (2009). The present and future burden of urinary bladder cancer in the world. World J Urol.

[35] Rubben H, Lutzeyer W, Fischer N, Deutz F, Lagrange W, Giani G (1988). Natural history and treatment of low and high risk superficial bladder tumors. J Urol.

[36] Siegel R, Naishadham D, Jemal A (2013). Cancer statistics, 2013. CA Cancer J Clin.

[37] Sun M, Thuret R, Abdollah F, Lughezzani G, Schmitges J, Tian Z, Shariat SF, Montorsi F, Patard JJ, Perrotte P, Karakiewicz PI (2011). Age-adjusted incidence, mortality, and survival rates of stage-specific renal cell carcinoma in North America: a trend analysis. Eur Urol.

[38] Chin AI, Lam JS, Figlin RA, Belldegrun AS (2006). Surveillance strategies for renal cell carcinoma patients following nephrectomy. Reviews in urology.

[39] Marchesi I, Giordano A, Bagella L (2014). Roles of enhancer of zeste homolog 2: from skeletal muscle differentiation to rhabdomyosarcoma carcinogenesis. Cell cycle.

[40] Mu Z, Li H, Fernandez SV, Alpaugh KR, Zhang R, Cristofanilli M (2013). EZH2 knockdown suppresses the growth and invasion of human inflammatory breast cancer cells. Journal of experimental & clinical cancer research.

[41] Zhang J, Chen L, Han L, Shi Z, Zhang J, Pu P, Kang C (2015). EZH2 is a negative prognostic factor and exhibits pro-oncogenic activity in glioblastoma. Cancer Lett.

[42] Zhang X, Zhang Y, Liu X, Liu T, Li P, Du L, Yang Y, Wang L, Wang C (2016). Nested quantitative PCR approach for urinary cell-free EZH2 mRNA and its potential clinical application in bladder cancer. Int J Cancer.

[43] Cao R, Wang L, Wang H, Xia L, Erdjument-Bromage H, Tempst P, Jones RS, Zhang Y (2002). Role of histone H3 lysine 27 methylation in Polycomb-group silencing. Science.

[44] Chang CJ, Yang JY, Xia W, Chen CT, Xie X, Chao CH, Woodward WA, Hsu JM, Hortobagyi GN, Hung MC (2011). EZH2 promotes expansion of breast tumor initiating cells through activation of RAF1-beta-catenin signaling. Cancer Cell.

[45] Lee TI, Jenner RG, Boyer LA, Guenther MG, Levine SS, Kumar RM, Chevalier B, Johnstone SE, Cole MF, Isono K, Koseki H, Fuchikami T, Abe K (2006). Control of developmental regulators by Polycomb in human embryonic stem cells. Cell.

[46] Boyer LA, Plath K, Zeitlinger J, Brambrink T, Medeiros LA, Lee TI, Levine SS, Wernig M, Tajonar A, Ray MK, Bell GW, Otte AP, Vidal M (2006). Polycomb complexes repress developmental regulators in murine embryonic stem cells. Nature.

[47] Ezhkova E, Pasolli HA, Parker JS, Stokes N, Su IH, Hannon G, Tarakhovsky A, Fuchs E (2009). Ezh2 orchestrates gene expression for the stepwise differentiation of tissue-specific stem cells. Cell.

[48] Schwarz D, Varum S, Zemke M, Scholer A, Baggiolini A, Draganova K, Koseki H, Schubeler D, Sommer L (2014). Ezh2 is required for neural crest-derived cartilage and bone formation. Development.

[49] Du J, Li L, Ou Z, Kong C, Zhang Y, Dong Z, Zhu S, Jiang H, Shao Z, Huang B, Lu J (2012). FOXC1, a target of polycomb, inhibits metastasis of breast cancer cells. Breast cancer research and treatment.

[50] Cao Q, Yu J, Dhanasekaran SM, Kim JH, Mani RS, Tomlins SA, Mehra R, Laxman B, Cao X, Yu J, Kleer CG, Varambally S, Chinnaiyan AM (2008). Repression of E-cadherin by the polycomb group protein EZH2 in cancer. Oncogene.

[51] Tiwari N, Tiwari VK, Waldmeier L, Balwierz PJ, Arnold P, Pachkov M, Meyer-Schaller N, Schubeler D, van Nimwegen E, Christofori G (2013). Sox4 is a master regulator of epithelial-mesenchymal transition by controlling Ezh2 expression and epigenetic reprogramming. Cancer Cell.

[52] Liu X, Wang C, Chen Z, Jin Y, Wang Y, Kolokythas A, Dai Y, Zhou X (2011). MicroRNA-138 suppresses epithelial-mesenchymal transition in squamous cell carcinoma cell lines. The Biochemical journal.

[53] Liu L, Xu Z, Zhong L, Wang H, Jiang S, Long Q, Xu J, Guo J (2016). Enhancer of zeste homolog 2 (EZH2) promotes tumour cell migration and invasion via epigenetic repression of E-cadherin in renal cell carcinoma. BJU Int.

[54] Nolan KD, Franco OE, Hance MW, Hayward SW, Isaacs JS (2015). Tumor-secreted Hsp90 subverts polycomb function to drive prostate tumor growth and invasion. The Journal of biological chemistry.

[55] Tong ZT, Cai MY, Wang XG, Kong LL, Mai SJ, Liu YH, Zhang HB, Liao YJ, Zheng F, Zhu W, Liu TH, Bian XW, Guan XY (2012). EZH2 supports nasopharyngeal carcinoma cell aggressiveness by forming a co-repressor complex with HDAC1/HDAC2 and Snail to inhibit E-cadherin. Oncogene.

[56] Chen J, Xu H, Zou X, Wang J, Zhu Y, Chen H, Shen B, Deng X, Zhou A, Chin YE, Rauscher FJ, Peng C, Hou Z (2014). Snail recruits Ring1B to mediate transcriptional repression and cell migration in pancreatic cancer cells. Cancer Res.

[57] Sun J, Zheng G, Gu Z, Guo Z (2015). MiR-137 inhibits proliferation and angiogenesis of human glioblastoma cells by targeting EZH2. Journal of neuro-oncology.

[58] Mitic T, Caporali A, Floris I, Meloni M, Marchetti M, Urrutia R, Angelini GD, Emanueli C (2015). EZH2 modulates angiogenesis in vitro and in a mouse model of limb ischemia. Molecular therapy.

[59] Shi B, Liang J, Yang X, Wang Y, Zhao Y, Wu H, Sun L, Zhang Y, Chen Y, Li R, Zhang Y, Hong M, Shang Y (2007). Integration of estrogen and Wnt signaling circuits by the polycomb group protein EZH2 in breast cancer cells. Molecular and cellular biology.

[60] Jung HY, Jun S, Lee M, Kim HC, Wang X, Ji H, McCrea PD, Park JI (2013). PAF and EZH2 induce Wnt/beta-catenin signaling hyperactivation. Molecular cell.

[61] Lee ST, Li Z, Wu Z, Aau M, Guan P, Karuturi RK, Liou YC, Yu Q (2011). Context-specific regulation of NF-kappaB target gene expression by EZH2 in breast cancers. Molecular cell.

[62] Gonzalez ME, Moore HM, Li X, Toy KA, Huang W, Sabel MS, Kidwell KM, Kleer CG (2014). EZH2 expands breast stem cells through activation of NOTCH1 signaling. Proc Natl Acad Sci U S A.

[63] Xu K, Wu ZJ, Groner AC, He HH, Cai C, Lis RT, Wu X, Stack EC, Loda M, Liu T, Xu H, Cato L, Thornton JE (2012). EZH2 oncogenic activity in castration-resistant prostate cancer cells is Polycomb-independent. Science.

[64] Kim E, Kim M, Woo DH, Shin Y, Shin J, Chang N, Oh YT, Kim H, Rheey J, Nakano I, Lee C, Joo KM, Rich JN (2013). Phosphorylation of EZH2 activates STAT3 signaling via STAT3 methylation and promotes tumorigenicity of glioblastoma stem-like cells. Cancer Cell.

[65] Lee JM, Lee JS, Kim H, Kim K, Park H, Kim JY, Lee SH, Kim IS, Kim J, Lee M, Chung CH, Seo SB, Yoon JB (2012). EZH2 generates a methyl degron that is recognized by the DCAF1/DDB1/CUL4 E3 ubiquitin ligase complex. Molecular cell.

[66] Berezovska OP, Glinskii AB, Yang Z, Li XM, Hoffman RM, Glinsky GV (2006). Essential role for activation of the Polycomb group (PcG) protein chromatin silencing pathway in metastatic prostate cancer. Cell cycle.

[67] Saramaki OR, Tammela TL, Martikainen PM, Vessella RL, Visakorpi T (2006). The gene for polycomb group protein enhancer of zeste homolog 2 (EZH2) is amplified in late-stage prostate cancer. Genes, chromosomes & cancer.

[68] Hoffmann MJ, Engers R, Florl AR, Otte AP, Muller M, Schulz WA (2007). Expression changes in EZH2, but not in BMI-1, SIRT1, DNMT1 or DNMT3B are associated with DNA methylation changes in prostate cancer. Cancer biology & therapy.

[69] Bracken AP, Pasini D, Capra M, Prosperini E, Colli E, Helin K (2003). EZH2 is downstream of the pRB-E2F pathway, essential for proliferation and amplified in cancer. The EMBO journal.

[70] Tang X, Milyavsky M, Shats I, Erez N, Goldfinger N, Rotter V (2004). Activated p53 suppresses the histone methyltransferase EZH2 gene. Oncogene.

[71] Lu W, Liu S, Li B, Xie Y, Izban MG, Ballard BR, Sathyanarayana SA, Adunyah SE, Matusik RJ, Chen Z (2016). SKP2 loss destabilizes EZH2 by promoting TRAF6-mediated ubiquitination to suppress prostate cancer. Oncogene.

[72] Jiang X, Zhang C, Qi S, Guo S, Chen Y, Du E, Zhang H, Wang X, Liu R, Qiao B, Yang K, Zhang Z, Xu Y (2016). Elevated expression of ZNF217 promotes prostate cancer growth by restraining ferroportin-conducted iron egress. Oncotarget.

[73] Chen K, Xiao H, Zeng J, Yu G, Zhou H, Huang C, Yao W, Xiao W, Hu J, Guan W, Wu L, Huang J, Huang Q (2016). Alternative splicing of EZH2 pre-mRNA by SF3B3 contributes to the tumorigenic potential of renal cancer. Clin Cancer Res.

[74] Wang D, Ding L, Wang L, Zhao Y, Sun Z, Karnes RJ, Zhang J, Huang H (2015). LncRNA MALAT1 enhances oncogenic activities of EZH2 in castration-resistant prostate cancer. Oncotarget.

[75] Chen H, Tu SW, Hsieh JT (2005). Down-regulation of human DAB2IP gene expression mediated by polycomb Ezh2 complex and histone deacetylase in prostate cancer. The Journal of biological chemistry.

[76] Min J, Zaslavsky A, Fedele G, McLaughlin SK, Reczek EE, De Raedt T, Guney I, Strochlic DE, Macconaill LE, Beroukhim R, Bronson RT, Ryeom S, Hahn WC (2010). An oncogene-tumor suppressor cascade drives metastatic prostate cancer by coordinately activating Ras and nuclear factor-kappaB. Nature medicine.

[77] Yu J, Cao Q, Mehra R, Laxman B, Yu J, Tomlins SA, Creighton CJ, Dhanasekaran SM, Shen R, Chen G, Morris DS, Marquez VE, Shah RB (2007). Integrative genomics analysis reveals silencing of beta-adrenergic signaling by polycomb in prostate cancer. Cancer cell.

[78] Beke L, Nuytten M, Van Eynde A, Beullens M, Bollen M (2007). The gene encoding the prostatic tumor suppressor PSP94 is a target for repression by the Polycomb group protein EZH2. Oncogene.

[79] Yu J, Cao Q, Yu J, Wu L, Dallol A, Li J, Chen G, Grasso C, Cao X, Lonigro RJ, Varambally S, Mehra R, Palanisamy N (2010). The neuronal repellent SLIT2 is a target for repression by EZH2 in prostate cancer. Oncogene.

[80] Zhang Y, Tong T (2014). FOXA1 antagonizes EZH2-mediated CDKN2A repression in carcinogenesis. Biochemical and biophysical research communications.

[81] Shin YJ, Kim JH (2012). The role of EZH2 in the regulation of the activity of matrix metalloproteinases in prostate cancer cells. PloS one.

[82] Jia J, Li F, Tang XS, Xu S, Gao Y, Shi Q, Guo W, Wang X, He D, Guo P (2016). Long noncoding RNA DANCR promotes invasion of prostate cancer through epigenetically silencing expression of TIMP2/3. Oncotarget.

[83] Dahlman A, Edsjo A, Hallden C, Persson JL, Fine SW, Lilja H, Gerald W, Bjartell A (2010). Effect of androgen deprivation therapy on the expression of prostate cancer biomarkers MSMB and MSMB-binding protein CRISP3. Prostate cancer and prostatic diseases.

[84] Prensner JR, Iyer MK, Balbin OA, Dhanasekaran SM, Cao Q, Brenner JC, Laxman B, Asangani IA, Grasso CS, Kominsky HD, Cao X, Jing X, Wang X (2011). Transcriptome sequencing across a prostate cancer cohort identifies PCAT-1, an unannotated lincRNA implicated in disease progression. Nat Biotechnol.

[85] Ren G, Baritaki S, Marathe H, Feng J, Park S, Beach S, Bazeley PS, Beshir AB, Fenteany G, Mehra R, Daignault S, Al-Mulla F, Keller E (2012). Polycomb protein EZH2 regulates tumor invasion via the transcriptional repression of the metastasis suppressor RKIP in breast and prostate cancer. Cancer Res.

[86] Takayama K, Suzuki T, Tsutsumi S, Fujimura T, Urano T, Takahashi S, Homma Y, Aburatani H, Inoue S (2015). RUNX1, an androgen- and EZH2-regulated gene, has differential roles in AR-dependent and -independent prostate cancer. Oncotarget.

[87] Erdmann K, Kaulke K, Rieger C, Salomo K, Wirth MP, Fuessel S (2016). MiR-26a and miR-138 block the G1/S transition by targeting the cell cycle regulating network in prostate cancer cells. J Cancer Res Clin Oncol.

[88] Koh CM, Iwata T, Zheng Q, Bethel C, Yegnasubramanian S, De Marzo AM (2011). Myc enforces overexpression of EZH2 in early prostatic neoplasia via transcriptional and post-transcriptional mechanisms. Oncotarget.

[89] Martinez-Fernandez M, Rubio C, Segovia C, Lopez-Calderon FF, Duenas M, Paramio JM (2015). EZH2 in Bladder Cancer, a Promising Therapeutic Target. Int J Mol Sci.

[90] Cha TL, Zhou BP, Xia W, Wu Y, Yang CC, Chen CT, Ping B, Otte AP, Hung MC (2005). Akt-mediated phosphorylation of EZH2 suppresses methylation of lysine 27 in histone H3. Science.

[91] Hinz S, Kempkensteffen C, Weikert S, Schostak M, Schrader M, Miller K, Christoph F (2007). EZH2 polycomb transcriptional repressor expression correlates with methylation of the APAF-1 gene in superficial transitional cell carcinoma of the bladder. Tumour Biol.

[92] Luo M, Li Z, Wang W, Zeng Y, Liu Z, Qiu J (2013). Long non-coding RNA H19 increases bladder cancer metastasis by associating with EZH2 and inhibiting E-cadherin expression. Cancer Lett.

[93] Santos M, Martinez-Fernandez M, Duenas M, Garcia-Escudero R, Alfaya B, Villacampa F, Saiz-Ladera C, Costa C, Oteo M, Duarte J, Martinez V, Gomez-Rodriguez MJ, Martin ML (2014). In vivo disruption of an Rb-E2F-Ezh2 signaling loop causes bladder cancer. Cancer Res.

[94] Wu X, Liu D, Tao D, Xiang W, Xiao X, Wang M, Wang L, Luo G, Li Y, Zeng F, Jiang G (2016). BRD4 Regulates EZH2 Transcription through Upregulation of C-MYC and Represents a Novel Therapeutic Target in Bladder Cancer. Molecular cancer therapeutics.

[95] Calin GA, Croce CM (2006). MicroRNA signatures in human cancers. Nature reviews Cancer.

[96] Guo Y, Ying L, Tian Y, Yang P, Zhu Y, Wang Z, Qiu F, Lin J (2013). miR-144 downregulation increases bladder cancer cell proliferation by targeting EZH2 and regulating Wnt signaling. The FEBS journal.

[97] Friedman JM, Liang G, Liu CC, Wolff EM, Tsai YC, Ye W, Zhou X, Jones PA (2009). The putative tumor suppressor microRNA-101 modulates the cancer epigenome by repressing the polycomb group protein EZH2. Cancer research.

[98] Tzatsos A, Paskaleva P, Lymperi S, Contino G, Stoykova S, Chen Z, Wong KK, Bardeesy N (2011). Lysine-specific demethylase 2B (KDM2B)-let-7-enhancer of zester homolog 2 (EZH2) pathway regulates cell cycle progression and senescence in primary cells. The Journal of biological chemistry.

[99] Kottakis F, Polytarchou C, Foltopoulou P, Sanidas I, Kampranis SC, Tsichlis PN (2011). FGF-2 regulates cell proliferation, migration, and angiogenesis through an NDY1/KDM2B-miR-101-EZH2 pathway. Molecular cell.

[100] Tsai MC, Manor O, Wan Y, Mosammaparast N, Wang JK, Lan F, Shi Y, Segal E, Chang HY (2010). Long noncoding RNA as modular scaffold of histone modification complexes. Science.

[101] Zhang S, Zhong G, He W, Yu H, Huang J, Lin T (2016). lncRNA Up-Regulated in Nonmuscle Invasive Bladder Cancer Facilitates Tumor Growth and Acts as a Negative Prognostic Factor of Recurrence. J Urol.

[102] He W, Cai Q, Sun F, Zhong G, Wang P, Liu H, Luo J, Yu H, Huang J, Lin T (2013). linc-UBC1 physically associates with polycomb repressive complex 2 (PRC2) and acts as a negative prognostic factor for lymph node metastasis and survival in bladder cancer. Biochimica et biophysica acta.

[103] Liu L, Xu Z, Zhong L, Wang H, Jiang S, Long Q, Xu J, Guo J (2013). Prognostic value of EZH2 expression and activity in renal cell carcinoma: a prospective study. PLoS One.

[104] Shen Y, Guo X, Wang Y, Qiu W, Chang Y, Zhang A, Duan X (2012). Expression and significance of histone H3K27 demethylases in renal cell carcinoma. BMC cancer.

[105] Lee HW, Choe M (2012). Expression of EZH2 in renal cell carcinoma as a novel prognostic marker. Pathology international.

[106] Avissar-Whiting M, Koestler DC, Houseman EA, Christensen BC, Kelsey KT, Marsit CJ (2011). Polycomb group genes are targets of aberrant DNA methylation in renal cell carcinoma. Epigenetics.

[107] Wagener N, Macher-Goeppinger S, Pritsch M, Husing J, Hoppe-Seyler K, Schirmacher P, Pfitzenmaier J, Haferkamp A, Hoppe-Seyler F, Hohenfellner M (2010). Enhancer of zeste homolog 2 (EZH2) expression is an independent prognostic factor in renal cell carcinoma. BMC cancer.

[108] Wang Y, Chen Y, Geng H, Qi C, Liu Y, Yue D (2015). Overexpression of YB1 and EZH2 are associated with cancer metastasis and poor prognosis in renal cell carcinomas. Tumour Biol.

[109] Hirata H, Hinoda Y, Shahryari V, Deng G, Nakajima K, Tabatabai ZL, Ishii N, Dahiya R (2015). Long Noncoding RNA MALAT1 Promotes Aggressive Renal Cell Carcinoma through Ezh2 and Interacts with miR-205. Cancer Res.

[110] Wu Y, Liu J, Zheng Y, You L, Kuang D, Liu T (2014). Suppressed expression of long non-coding RNA HOTAIR inhibits proliferation and tumourigenicity of renal carcinoma cells. Tumour Biol.

[111] Sakurai T, Bilim VN, Ugolkov AV, Yuuki K, Tsukigi M, Motoyama T, Tomita Y (2012). The enhancer of zeste homolog 2 (EZH2), a potential therapeutic target, is regulated by miR-101 in renal cancer cells. Biochemical and biophysical research communications.

[112] Liang J, Zhang Y, Jiang G, Liu Z, Xiang W, Chen X, Chen Z, Zhao J (2013). MiR-138 induces renal carcinoma cell senescence by targeting EZH2 and is downregulated in human clear cell renal cell carcinoma. Oncol Res.

[113] Du Z, Li L, Huang X, Jin J, Huang S, Zhang Q, Tao Q (2016). The epigenetic modifier CHD5 functions as a novel tumor suppressor for renal cell carcinoma and is predominantly inactivated by promoter CpG methylation. Oncotarget.

[114] Yuan JB, Yang LY, Tang ZY, Zu XB, Qi L (2012). Down-regulation of EZH2 by RNA interference inhibits proliferation and invasion of ACHN cells via the Wnt/beta- catenin pathway. Asian Pacific journal of cancer prevention.

[115] Wu C, Jin X, Yang J, Yang Y, He Y, Ding L, Pan Y, Chen S, Jiang J, Huang H (2016). Inhibition of EZH2 by chemo- and radiotherapy agents and small molecule inhibitors induces cell death in castration-resistant prostate cancer. Oncotarget.

[116] Wu Y, Yu J, Liu Y, Yuan L, Yan H, Jing J, Xu G (2014). Delivery of EZH2-shRNA with mPEG-PEI nanoparticles for the treatment of prostate cancer in vitro. International journal of molecular medicine.

[117] Zhang Q, Zhao W, Ye C, Zhuang J, Chang C, Li Y, Huang X, Shen L, Li Y, Cui Y, Song J, Shen B, Eliaz I (2015). Honokiol inhibits bladder tumor growth by suppressing EZH2/miR-143 axis. Oncotarget.

[118] Wang HF, Yang H, Hu LB, Lei YH, Qin Y, Li J, Bi CW, Wang JS, Huo Q (2014). Effect of siRNA targeting EZH2 on cell viability and apoptosis of bladder cancer T24 cells. Genetics and molecular research.

[119] Sun F, Lee L, Zhang Z, Wang X, Yu Q, Duan X, Chan E (2015). Preclinical pharmacokinetic studies of 3-deazaneplanocin A, a potent epigenetic anticancer agent, and its human pharmacokinetic prediction using GastroPlus. European journal of pharmaceutical sciences.

[120] Zhang R, Wang R, Chang H, Wu F, Liu C, Deng D, Fan W (2012). Downregulation of Ezh2 expression by RNA interference induces cell cycle arrest in the G0/G1 phase and apoptosis in U87 human glioma cells. Oncol Rep.

[121] Tan J, Yang X, Zhuang L, Jiang X, Chen W, Lee PL, Karuturi RK, Tan PB, Liu ET, Yu Q (2007). Pharmacologic disruption of Polycomb-repressive complex 2-mediated gene repression selectively induces apoptosis in cancer cells. Genes & development.

[122] Hibino S, Saito Y, Muramatsu T, Otani A, Kasai Y, Kimura M, Saito H (2014). Inhibitors of enhancer of zeste homolog 2 (EZH2) activate tumor-suppressor microRNAs in human cancer cells. Oncogenesis.

[123] Fiskus W, Wang Y, Sreekumar A, Buckley KM, Shi H, Jillella A, Ustun C, Rao R, Fernandez P, Chen J, Balusu R, Koul S, Atadja P (2009). Combined epigenetic therapy with the histone methyltransferase EZH2 inhibitor 3-deazaneplanocin A and the histone deacetylase inhibitor panobinostat against human AML cells. Blood.

[124] Girard N, Bazille C, Lhuissier E, Benateau H, Llombart-Bosch A, Boumediene K, Bauge C (2014). 3-Deazaneplanocin A (DZNep), an inhibitor of the histone methyltransferase EZH2, induces apoptosis and reduces cell migration in chondrosarcoma cells. PLoS One.

[125] Hayden A, Johnson PW, Packham G, Crabb SJ (2011). S-adenosylhomocysteine hydrolase inhibition by 3-deazaneplanocin A analogues induces anti-cancer effects in breast cancer cell lines and synergy with both histone deacetylase and HER2 inhibition. Breast cancer research and treatment.

[126] Kikuchi J, Takashina T, Kinoshita I, Kikuchi E, Shimizu Y, Sakakibara-Konishi J, Oizumi S, Marquez VE, Nishimura M, Dosaka-Akita H (2012). Epigenetic therapy with 3-deazaneplanocin A, an inhibitor of the histone methyltransferase EZH2, inhibits growth of non-small cell lung cancer cells. Lung cancer.

[127] Miranda TB, Cortez CC, Yoo CB, Liang G, Abe M, Kelly TK, Marquez VE, Jones PA (2009). DZNep is a global histone methylation inhibitor that reactivates developmental genes not silenced by DNA methylation. Molecular cancer therapeutics.

[128] Hsieh YY, Lo HL, Yang PM (2016). EZH2 inhibitors transcriptionally upregulate cytotoxic autophagy and cytoprotective unfolded protein response in human colorectal cancer cells. American journal of cancer research.

[129] McCabe MT, Ott HM, Ganji G, Korenchuk S, Thompson C, Van Aller GS, Liu Y, Graves AP, Della Pietra A, Diaz E, LaFrance LV, Mellinger M, Duquenne C (2012). EZH2 inhibition as a therapeutic strategy for lymphoma with EZH2-activating mutations. Nature.

[130] Verma SK, Tian X, LaFrance LV, Duquenne C, Suarez DP, Newlander KA, Romeril SP, Burgess JL, Grant SW, Brackley JA, Graves AP, Scherzer DA, Shu A (2012). Identification of Potent, Selective, Cell-Active Inhibitors of the Histone Lysine Methyltransferase EZH2. ACS medicinal chemistry letters.

[131] Qi W, Chan H, Teng L, Li L, Chuai S, Zhang R, Zeng J, Li M, Fan H, Lin Y, Gu J, Ardayfio O, Zhang JH (2012). Selective inhibition of Ezh2 by a small molecule inhibitor blocks tumor cells proliferation. Proc Natl Acad Sci U S A.

[132] Konze KD, Ma A, Li F, Barsyte-Lovejoy D, Parton T, Macnevin CJ, Liu F, Gao C, Huang XP, Kuznetsova E, Rougie M, Jiang A, Pattenden SG (2013). An orally bioavailable chemical probe of the Lysine Methyltransferases EZH2 and EZH1. ACS chemical biology.

[133] Knutson SK, Warholic NM, Wigle TJ, Klaus CR, Allain CJ, Raimondi A, Porter Scott M, Chesworth R, Moyer MP, Copeland RA, Richon VM, Pollock RM, Kuntz KW (2013). Durable tumor regression in genetically altered malignant rhabdoid tumors by inhibition of methyltransferase EZH2. Proc Natl Acad Sci U S A.

[134] Kim W, Bird GH, Neff T, Guo G, Kerenyi MA, Walensky LD, Orkin SH (2013). Targeted disruption of the EZH2-EED complex inhibits EZH2-dependent cancer. Nature chemical biology.

[135] Gibaja V, Shen F, Harari J, Korn J, Ruddy D, Saenz-Vash V, Zhai H, Rejtar T, Paris CG, Yu Z, Lira M, King D, Qi W (2016). Development of secondary mutations in wild-type and mutant EZH2 alleles cooperates to confer resistance to EZH2 inhibitors. Oncogene.

[136] Kirk JS, Schaarschuch K, Dalimov Z, Lasorsa E, Ku S, Ramakrishnan S, Hu Q, Azabdaftari G, Wang J, Pili R, Ellis L (2015). Top2a identifies and provides epigenetic rationale for novel combination therapeutic strategies for aggressive prostate cancer. Oncotarget.

[137] Kondo Y, Shen L, Cheng AS, Ahmed S, Boumber Y, Charo C, Yamochi T, Urano T, Furukawa K, Kwabi-Addo B, Gold DL, Sekido Y, Huang TH (2008). Gene silencing in cancer by histone H3 lysine 27 trimethylation independent of promoter DNA methylation. Nature genetics.

[138] Chen H, Rossier C, Antonarakis SE (1996). Cloning of a human homolog of the Drosophila enhancer of zeste gene (EZH2) that maps to chromosome 21q22.2. Genomics.

